# Polymorphisms of GLP-1 Receptor Gene and Response to GLP-1 Analogue in Patients with Poorly Controlled Type 2 Diabetes

**DOI:** 10.1155/2015/176949

**Published:** 2015-02-15

**Authors:** Chia-Hung Lin, Yun-Shien Lee, Yu-Yao Huang, Sheng-Hwu Hsieh, Zih-Syuan Chen, Chi-Neu Tsai

**Affiliations:** ^1^Division of Endocrinology and Metabolism, Department of Internal Medicine, Chang Gung Memorial Hospital, Taoyuan 333, Taiwan; ^2^Graduate Institute of Clinical Medical Sciences, Chang Gung University, Taoyuan 333, Taiwan; ^3^Department of Biotechnology, Ming Chuan University, Taoyuan 333, Taiwan; ^4^Genomic Medicine Research Core Laboratory, Chang Gung Memorial Hospital, Taoyuan 333, Taiwan

## Abstract

*Aim*. The relationship between genetic polymorphisms of the glucagon-like peptide-1 (GLP-1) receptor (*GLP1R*) gene and unresponsiveness to GLP-1 analogue treatment in patients with poorly controlled type 2 diabetes mellitus (DM) is unclear. *Methods*. Thirty-six patients with poorly controlled type 2 DM were enrolled and they received six days of continuous subcutaneous insulin infusion for this study. After the normalization of blood glucose in the first 3 days, the patients then received a combination therapy with injections of the GLP-1 analogue, exenatide, for another 3 days. All 13 exons and intron-exon boundaries of the *GLP1R* gene were amplified to investigate the association. *Results*. The short tandem repeat at 8GA/7GA (rs5875654) had complete linkage disequilibrium (LD, with *r* ^2^ = 1) with single nucleotide polymorphism (SNP) rs761386. Quantitative trait loci analysis of *GLP1R* gene variation with clinical response of GLP1 analogue showed the missense rs3765467 and rs761386 significantly associated with changes in the standard deviation of plasma glucose (SDPG_baseline_ − SDPG_treatment with GLP-1 analogue_) (*P* = 0.041 and 0.019, resp.). The reported *P* values became insignificant after multiple testing adjustments. *Conclusion*. The variable response to the GLP-1 analogue was not statistically correlated with polymorphisms of the *GLP1R* gene in patients with poorly controlled type 2 DM.

## 1. Introduction

Glucagon-like peptide-1 (GLP-1) is secreted from the enteroendocrine L cells of the intestinal mucosa and is released into the portal circulation in response to meal ingestion [1] through posttranslational processing of proglucagon by prohormone convertase-1 in its secretory cells [2]. GLP-1 enhances insulin secretion and inhibits glucagon release in a glucose-dependent manner, prompting the development of GLP-1-based therapies for the treatment of diabetes [3]. GLP-1-based diabetes therapies affect glucose control through several mechanisms, including slowed gastric emptying, regulation of postprandial glucagon, reduction of food intake, and enhancement of glucose-dependent insulin secretion without the risk of hypoglycemia [4]. However, the clinical responsiveness to GLP-1 analogues varies among patients with type 2 diabetes mellitus [5], which suggests that genetic factors may be crucial in the pharmacological responsiveness of these patients. In order to establish the correct treatment protocols in clinical practice and taking into consideration the high cost of these new drugs, it is important to clarify this critical issue in patients with type 2 diabetes mellitus.

Among genetic variants, the diabetes-associated variants in* TCF7L2* (rs7903146) and* WFS1* (rs10010131) have been shown to affect the response to exogenous GLP-1, while variants in* KCNQ1* (rs151290, rs2237892, and rs2237895) have been reported to alter endogenous GLP-1 secretion [6–8]. However, a validation study showed no effect regarding variants in* TCF7L2*,* KCNQ1*, and* WFS1* on GLP-1 concentrations after a standard 75 g oral glucose tolerance test (OGTT) or GLP-1-induced insulin secretion in healthy subjects without diabetes [9].

The glucagon-like peptide 1 receptor (GLP1R) specifically binds GLP-1 and related peptides with a lower affinity such as the gastric inhibitory polypeptide and glucagon [10]. The GLP1R is a member of the class B1 family of G protein-coupled receptors, and polar interactions (hydrogen bonds or salt bridges) between GLP1R and agonists have recently been predicted [11]. Some* GLP1R* gene polymorphisms have been found to be related to the strength of these interactions [12]. However, the relationship between these polymorphisms and the responsiveness to GLP-1 analogue treatment has yet to be explored. Pharmacogenetics has the potential to increase benefits and reduce side effects in patients whose drug responses are not average, and possibly to tailor treatments for these outliers [13]. A previous study reported that differences in the insulinotropic response to exogenous GLP-1 in healthy volunteers depended on the presence or absence of two common polymorphisms of the* GLP1R* gene [14]. However, the relationship between these single nucleotide polymorphisms (SNPs) and the effect of GLP-1 analogues in patients with type 2 diabetes mellitus has not yet been established.

Currently, GLP-1 analogues are most often used for patients with poorly controlled type 2 diabetes mellitus. However, the overall general control rate is not good which may be partially due to the complex etiology involved in type 2 diabetes mellitus [3]. Furthermore, the lack of normal beta cell secretary function is emphasized in modern practice. Therefore, the effect of GLP-1 analogues could be affected by various beta cell functions in patients with type 2 diabetes mellitus [3]. In order to study the effect of a GLP-1 analogue in patients with poorly controlled type 2 diabetes mellitus, we first optimized insulin therapy in this study. Continuous subcutaneous insulin infusion (CSII) or an insulin pump is a viable choice for patients with diabetes mellitus who require close-to-physiologic insulin treatment [15]. With insulin pump therapy provided during hospitalization it is possible to standardize the sugar control profile in patients with type 2 diabetes mellitus in a short period of time, thereby allowing for the further evaluation of the clinical response to GLP-1 analogues. To investigate the relationship between the SNPs of* GLP1R* and the effectiveness of GLP-1 analogue treatment in patients with type 2 diabetes mellitus, we performed exon resequencing of the* GLP1R* gene in patients with poorly controlled type 2 diabetes mellitus who were treated with a GLP-1 analogue in this study.

## 2. Materials and Methods

### 2.1. Patients

Thirty-six patients with type 2 diabetes were enrolled into this study from 2011 to 2013. The inclusion criteria were (a) age > 20 years; (b) diabetes mellitus diagnosed > 2 years; (c) A1C level of 8% to 12%; and (d) receiving premixed insulin twice daily with a total insulin daily dose of > 0.6 u/kg/day. The exclusion criteria were (a) recent history of drug or alcohol abuse; (b) sensitivity to analogous products; (c) serious cardiovascular disorders; (d) participation in another clinical investigation study; (e) ongoing influenza, autoimmune disease, or other metabolic disorders; and (f) pregnant or lactating women. This study was approved by the Institutional Review Board of the Chang Gung Memorial Hospital and registered with ClinicalTrials.gov (NCT01473147 and NCT02026024). Written informed consent was obtained from all subjects.

### 2.2. Study Protocol

All of the participants received a 6-day course of CSII intensive treatment during hospitalization. A finger-stick test was performed to examine premeal (AC) and 2-hour postmeal (PC) glucose levels after three meals in addition to bedtime and nocturnal glucose levels for a total of 8 measurements a day. The glucose level was normalized in the first 3 days, and the patients received a combined therapy with exenatide 5 *μ*g twice daily for the remaining 3 days. The responsiveness to the GLP-1 analogue was evaluated by the standard deviation of plasma glucose (SDPG), mean amplitude of glycemic excursions (MAGE), and mean glucose compared to the baseline. The 75 g OGTT was performed at baseline and at the end of the study to assess the insulin sensitivity index and homeostasis model assessment-insulin resistance [16, 17]. We stopped pharmacological treatment for at least 12 hours (premixed insulin after the evening dose) before performing the 75 g OGTT at baseline. To eliminate the effect of ultra-short acting insulin, Aspart, in the use of CSII, the 75 g OGTT was performed 2 hours after CSII had been stopped (end of the study).

### 2.3. Continuous Subcutaneous Insulin Infusion (CSII)

The insulin regimen was switched from premixed insulin to CSII according to a previously described hospital-based protocol [18, 19]. In brief, the prepump total daily dose of insulin was used as the starting dose of CSII. Half of the dose was infused continuously as the basal dose, and the other half was divided for each meal as the bolus dose. The basal insulin dose was then titrated as precisely as 0.1 U per hour to maintain the blood glucose targets in the range of 90–140 mg/dL from bedtime throughout the nocturnal period, and at 70–140 mg/dL before each meal. The bolus insulin dose was titrated up or down carefully by 1 U for a fixed amount of carbohydrates to maintain the postprandial glucose range between 70 and 180 mg/dL. We found that using 50% of the total daily dose as the basal insulin dose was usually an overestimation among our patients. Therefore, we focused on reducing the basal infusion rate to prevent hypoglycemia and increased the bolus dosage for a fixed amount of carbohydrates during meals. All of the patients received an adequate adjustment based on this 3-day titration protocol. At the end of the study, the switch in treatment of twice-daily or multiple-daily injections in CSII was equal to the divided total daily insulin dose or the total daily basal dose and respective premeal bolus dose according to a recommended protocol [19]. The medical team included diabetologists, educators, and dieticians, who were all on call to manage any unexpected occurrences during hospitalization.

### 2.4. DNA Extraction and Direct Resequencing of the* GLP1R* Gene

Genomic DNA was extracted from the leukocytes of peripheral blood from the 36 patients according to the manufacturer's recommendations (Genomic DNA Extraction Kit, RBC Bioscience, Taiwan). PCR was performed to amplify the promoter, all 13 exons and intron-exon boundaries of the* GLP1R* gene (GenBank accession number AL035690) using specific primer sets and PCR conditions as described in Supplementary Table  1, in Supplementary Material available online at http://dx.doi.org/10.1155/2015/176949 [20]. All of PCR products were confirmed by electrophoresis on 1.5% agarose gels and directly sequenced using an automated sequencer ABI 377 (Applied Biosystems, Foster City, CA) to determine the DNA sequences.

### 2.5. Statistical Analysis

Differences between groups with regard to continuous variables were tested using Student's* t*-test. Differences in proportions were assessed using a chi-square test or Fisher's exact test, as appropriate. Multiple linear regression analyses using additive genetic models were performed to adjust baseline variables and conducted with SPSS 20 (IBM SPSS Inc., Chicago, IL, USA). Results were expressed as means ± standard error mean or percentage. The level of statistical significance was set at a *P* value of 0.05 or less. All statistical analyses were conducted using the MATLAB program, version R2013a (MathWorks Inc., Natick, MA, USA). All reported *P* values are unadjusted for multiple testing.

## 3. Results

### 3.1. Clinical Manifestations

The mean age, gender, mean BMI, duration of diabetes mellitus, and A1C levels are shown in Table 1. The mean glucose, SDPG, and MAGE were significantly decreased after GLP-1 analogue treatment (180.2 ± 5.4 versus 147.9 ± 3.8 mg/dL, *P* < 0.001; 65.1 ± 3.3 versus 50.7 ± 3.0 mg/dL, *P* < 0.001; and 123.9 ± 6.5 versus 98.8 ± 6.4 mg/dL, *P* = 0.001, resp.) (Table 1). The homeostasis model assessment-insulin resistance (HOMA-IR) and insulin sensitivity index (ISI) were not significantly different after GLP-1 analogue treatment (*P* = 0.343 and 0.570, resp.).

### 3.2. Associations of* GLP1R* Genetic Variations with Drug Responsiveness

**Figure 1:**
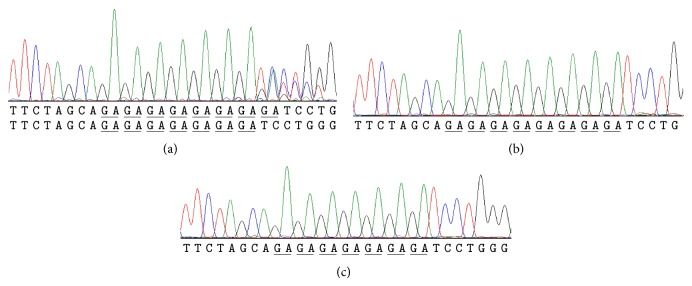
Experimental confirmation of the dinucleotide repeat polymorphism. A 2 bp deletion at chromosome 6: 39047037-39047052 was detected. (a) PCR direct sequencing chromatography trace. (b), (c) The PCR products were cloned, and at least 10 single colonies were analyzed by DNA sequencing. One plasmid contained the wild type sequence ((b), 8GA), whereas the other plasmids contained a deletion of GA ((c), 7GA). The underlined sequences indicate the one unit of the GA sequences.

**Figure 2:**
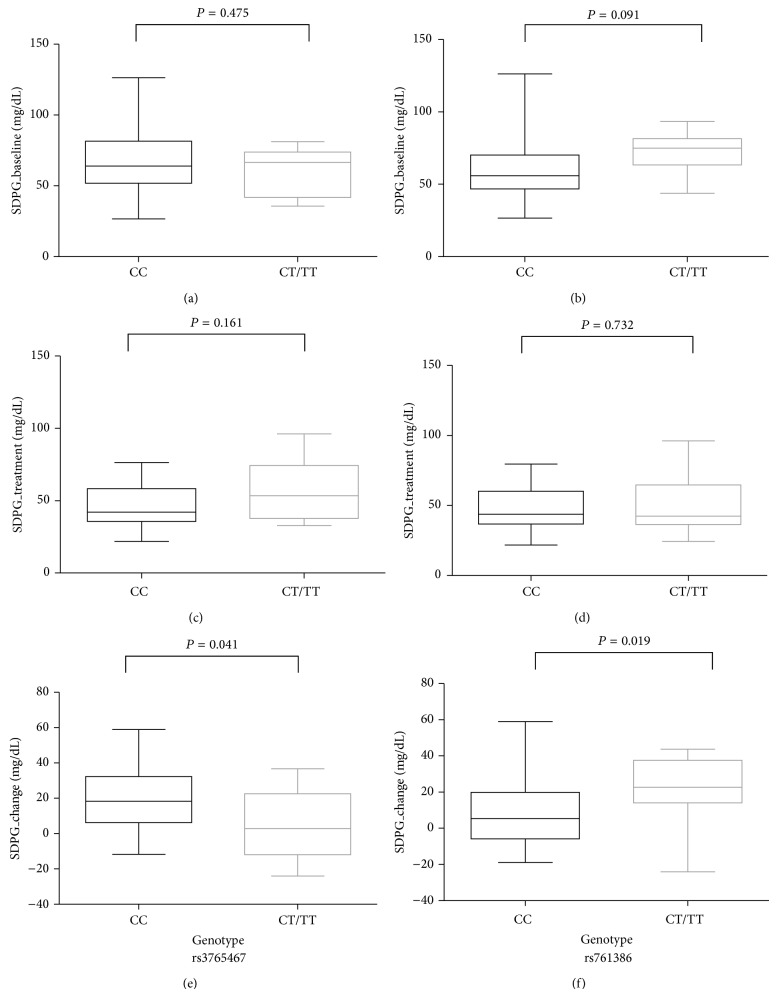
Quantitative trait loci analysis of* GLP1R* gene variations with clinical response to the GLP-1 analogue. (a)-(b) Baseline. (c)-(d) Treatment. (e)-(f) Change. SDPG_baseline_: the standard deviation of plasma glucose at baseline. SDPG_treatment  with  the  GLP-1  analogue_: the standard deviation of plasma glucose after treatment with GLP-1 analogue. SDPG change: the change of standard deviation of plasma glucose (SDPG_baseline_ − SDPG_treatment with the GLP-1 analogue_).

Nineteen SNPs around the exon region of the* GLP1R* gene were identified according to allele frequency (>0.2) in the 36 patients (Supplementary Figure  1). Among these 19 SNPs, we chose the reported missense SNP (rs3765467) [14] and the only one dinucleotide repeat polymorphism (rs5875654) for comparison. The rs5875654 was a short tandem repeat (STR) with 2-base-pair deletion of 8GA/7GA. The genotype of the 8GA/7GA variant was decomposed with the mixed sequence reader program [21] and further confirmed by PCR cloning following sequencing analysis (Figure 1). The rs5875654 and rs761386 SNPs showed complete linkage disequilibrium (LD, with *r* ^2^ = 1). The allele frequencies of these 3 missense and silent variants were depicted in Table 2. By analysis of the quantitative trait loci for other clinical variables, the two SNPs rs3765467 and rs761386 were found to be significantly associated with changes in the standard deviation of plasma glucose (SDPG_baseline_ − SDPG_treatment with GLP-1 analogue_) in the enrolled patients (*P* = 0.041 and 0.019, resp.) (Figure 2). The clinical characteristics of the subjects according to the recessive genotype subgroups are summarized in Supplementary Table  2 and all clinical variables were not significant between subgroups except for the sex distribution of rs3765467. However, the results remained the same and there was no change after adjusting for the sex variable. In particular, the T allele of rs3765467 and rs761386 was found to be associated with an opposite SDPG change (lower in rs3765467 and higher in rs761386) after GLP-1 analogue treatment. The association of the SDPG change with rs3765467 and rs761386 by multiple linear regression analyses using the additive genetic models with adjustment of age, sex, BMI, and glycemic states at baseline also demonstrated the same trend (Table 3).

The mean glucose and mean amplitude of glycemic excursions at baseline, treatment, and the change between the two time-points showed no significant differences between rs3765467 and rs761386 (Supplementary Figures  2 and 3). The association data of the remaining 16 common variants with each trait is shown in Supplementary Table  3.

**Figure 3:**
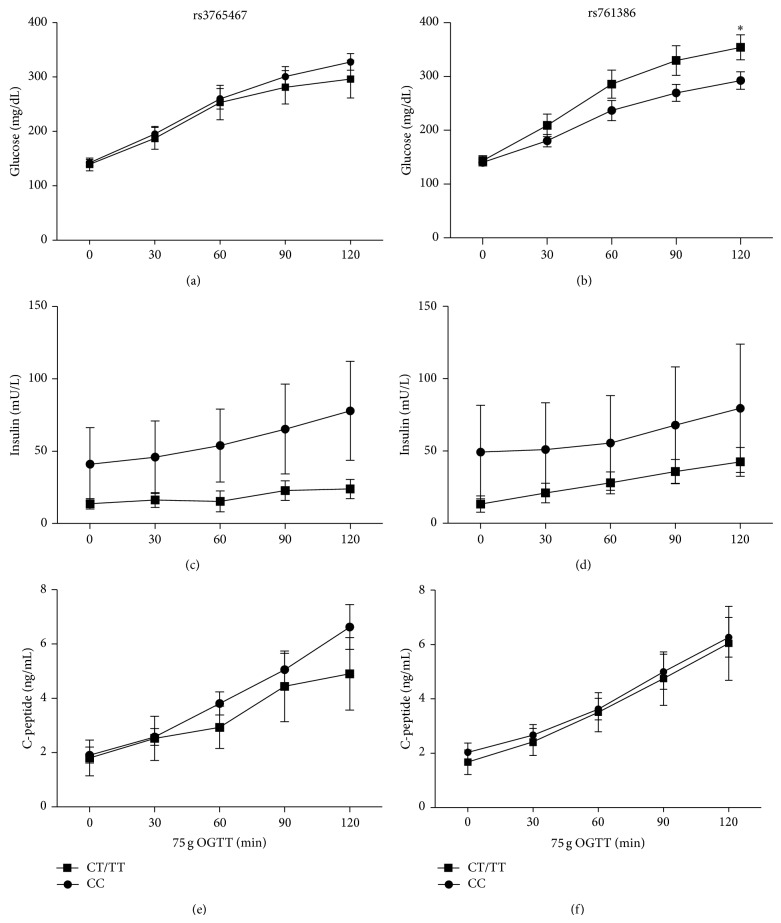
Effects of* GLP1R* genotype and time on the glucose (a)-(b), insulin (c)-(d), and C-peptide (e)-(f) levels of 75 g OGTT post GLP-1 analogue treatment. ^*^ *P* = 0.032 versus the time-matched genotype CC group.

The effects of* GLP1R* genotypes on glucose, insulin, and C-peptide concentrations during the 75 g OGTT after GLP-1 analogue treatment are shown in Figure 3. The (CT/TT) recessive model of rs761386 showed significantly higher glucose levels at 120 minutes of the 75 g OGTT (*P* = 0.032); however the insulin and C-peptide levels were not significantly different between the two genotypes throughout the OGTT for both rs3765467 and rs761386. The associations of the glucose, C-peptide, and insulin changes with rs3765467 and rs761386 by multiple linear regression analyses using the additive genetic models with and without adjustment of age, sex, BMI, and glycemic states at baseline showed no significance (Table 4). The reported *P* values became insignificant after multiple testing adjustments.

## 4. Discussion

To the best of our knowledge, this is the first study to reveal the relationship between genetic variations of* GLP1R* and the response to a GLP-1 analogue in patients with type 2 diabetes mellitus, although the number of enrolled cases in this study is limited. Based on our understanding of the characteristics of the GLP-1 analogue, exenatide, added to CSII during hospitalization, we could evaluate the real response to the GLP-1 analogue in patients with poorly controlled type 2 diabetes mellitus by excluding variable residual beta cell function. The combination of exenatide and insulin has previously been evaluated in clinical trials [22, 23]. In a placebo-controlled trial, exenatide added to insulin glargine reduced A1C by approximately 0.7% [23]. Another randomized trial examined the replacement of insulin with exenatide in patients with type 2 diabetes and found that glycemic control deteriorated in 38% (11 of 29) of the patients who received exenatide compared with 19% (3 of 16) of the patients who continued insulin [24]. The patients who lost glycemic control were more likely to have a longer duration of disease, lower C-peptide concentrations (suggesting less endogenous beta cell function), and larger insulin requirements at baseline. However, the combined use of basal-bolus or CSII and exenatide could maintain minimal beta cell function and potentiate the clinical effect of exenatide.

The 3-day conditioning period with CSII treatment is short compared to the time spent in the general outpatient therapy. But our protocol followed the suggestion of pump therapy in the previous report [19]. In this hospital based practice, we could simply focus on reducing the basal infusion rate to prevent hypoglycemia and increasing bolus insulin dosage for the fixed carbohydrate amount in meals. For the limitation of the total of one week of hospitalization, we could make use of the 3-day conditioning period with CSII treatment to efficiently detect the effect of GLP-1 analogue in these poorly controlled patients with type 2 DM.

Three SNPs have previously been associated with a response to infused GLP-1 or GLP-1 concentrations in response to an oral challenge (Table 5) [12, 14]. A previously published report showed that heterozygotes of the minor allele of rs3765467 were associated with an increase in GLP-1 response in healthy volunteers [14]. However, there were no significant differences in clinical response except for a lower SDPG change in the current study. Ethnic diversity and the characteristics of the participants may be reasons for this discrepancy.

As shown in Figures 2 and 3, a significant difference in SDPG change after GLP-1 analogue treatment was found between subgroups of genotype rs3765467 despite there being no significant differences in glucose, insulin, and C-peptide level on OGTT. The effects of GLP-1 analogue involve both beta cell and non-beta cell responses. Based on the results of this study, the variant of rs3765467 had an impact on SDPG change after GLP-1 analogue treatment favorably through the effect of non-beta cell related function, for example, glucagon suppression. The finding that there were no differences in glucose, insulin, and C-peptide levels in OGTT just reflected the lesser impact of this variant on the beta cell secretion.

The expression of a nonsynonymous SNP (rs367543060), which results in the substitution of methionine for threonine at position 149 of* GLP1R* in cell systems, has been documented to decrease binding affinity for GLP-1 and intracellular signaling after hormone receptor binding [12, 25]. The Thr149Met mutation was detected only in the proband among subjects with type 2 diabetes (1/791) but not in controls in a study from Japan [20]. Although the minor allele frequency data is not available at present, the variation of T149M of* GLP1R* was not detected in our enrolled patients. In the current study, the presence of the dinucleotide repeat polymorphism in the STR (8GA/7GA) of the* GLP1R* gene was nominally associated with altered glucose control with the use of a GLP-1 analogue. The significant SNPs in this study were located within intronic noncoding regions, and therefore the mechanisms of their actions remain elusive. Recent studies have reported that variants in* TCF7L2* (rs7903146) and* WFS1* (rs10010131), which have been shown to affect the response to exogenous GLP-1, and variants in* KCNQ1* (rs151290, rs2237892, and rs2237895), which have been demonstrated to alter endogenous GLP-1 secretion, are all identified in noncoding intron regions [6–9, 26]. Given that none of the chosen SNPs were located in coding regions, these genetic variants in* GLP1R* may affect gene expression but not the function of the gene product.

The actions of GLP-1 (primarily stimulation of insulin secretion and suppression of glucagon secretion) are mediated by binding to its cognate receptor. Exenatide, a GLP-1 receptor agonist, binds to the GLP-1 receptor with greater affinity than its natural ligand due to a nine-amino-acid COOH-terminal sequence that is absent in native GLP-1 [27]. The substitution of glycine for alanine at position eight of native GLP-1 has been reported to decrease its affinity for the receptor [28], suggesting that both N- and COOH-terminal ends of GLP-1 bind the receptor. The application of chimeric GLP-1/GIP peptides together with molecular modeling suggests that His^1^ of GLP-1 interacts with Asn^302^ of* GLP1R*, and that Thr^7^ of GLP-1 has close contact with a binding pocket formed by Ile^196^, Leu^232^, and Met^233^ of* GLP1R* [29]. The location of the STR related to the unresponsiveness of the GLP-1 analogue is around the coding region in Exons 9-10 responsible for the binding sites. Further studies assessing the function of gene regulation may help to clarify the relationship of this novel genetic variation and drug response.

One of the limitations of this study is the lack of data on the impact of long-term A1C control for the genetic variants of* GLP1R*. However, having well-controlled blood sugar management by CSII during the hospitalization period could help to further clarify the different pharmacological effects of the GLP-1 analogue in this type of patient. Although the reported *P* values became insignificant after multiple testing adjustments, the small sample size due to clinical difficulties in keeping patients hospitalized might not allow for such a statistical correction. Future large-scale studies aiming at elucidating the contribution of* GLP1R* genetic variations to GLP-1 analogue response will need to take into account the likelihood of the small effects of these variants on the quantitative traits to ensure that they are adequately powered to reproducibly determine such effects. While it is certainly possible that these variants had smaller effects on GLP-1 analogue-induced responses in this study, the clinical application of screening for genotype 7GA/7GA in rs5875654 and T/T in rs761386 could reveal which patients would be unresponsive to the GLP-1 analogue. It is important to develop approaches that help to effectively manage the use of expensive drugs in current modern incretin-based therapy of type 2 diabetes mellitus and to control unnecessary expenses.

## 5. Conclusion

The variable response to a GLP-1 analogue was not statistically correlated to the polymorphisms of the* GLP1R* gene in patients with poorly controlled type 2 diabetes mellitus.

## Supplementary Material

The promoter, all 13 exons and intron-exon boundaries of the *GLP1R* gene (GenBank accession number AL035690) were amplified by polymerase chain reaction (PCR) using specific primer sets in Supplementary Table 1. The Titanium *Taq* DNA Polymerase (Clontech Laboratories, Inc., Mountain View, CA, USA) was used in PCR. The PCR amplification conditions consisted of initial denaturation at 95°C for 5 minutes followed by 40 cycles at 95°C for 45 seconds, annealing temperature for 45 seconds and 72°C for 45 seconds, with one additional cycle at 72°C for 10 minutes.

## Figures and Tables

**Table 1:** Clinical variables of all participants with GLP-1 analogue.

Variable	Baseline	End of the study	*P*
*N*	36		
Age (years old)	52.8 ± 2.4		
Sex (female %)	44		
BMI (kg/m^2^)	28.87 ± 0.83		
DM duration (years)	11.6 ± 1.3		
A1C (%)	10.5 ± 0.2		
A1C (mmol/mol)	91.3 ± 2.5		
Fasting C-peptide (ng/mL)	1.68 ± 0.26	1.88 ± 0.27	0.353
Fasting insulin (mU/L)	31.85 ± 9.33	31.52 ± 15.50	0.971
Mean glucose (mg/dL)	180.2 ± 5.4	147.9 ± 3.8	<0.001^*^
SDPG (mg/dL)	65.1 ± 3.3	50.7 ± 3.0	<0.001^*^
MAGE (mg/dL)	123.9 ± 6.5	98.8 ± 6.4	0.001^*^
HOMA-IR	15.23 ± 4.32	11.88 ± 6.00	0.343
ISI	4.43 ± 1.03	5.03 ± 0.80	0.570

Data are expressed as mean ± standard error mean.

SDPG: standard deviation of plasma glucose.

MAGE: mean amplitude of glycemic excursions.

HOMA-IR: homeostasis model assessment-insulin resistance.

ISI: insulin sensitivity index.

^*^ *P* < 0.01 by paired-*t* test.

**Table 2:** The frequencies of missense and silent variants in 36 patients type 2 diabetic subjects.

Marker	Locus	Type/function	MAF	Genotype	Frequency
rs3765467	Exon 4	SNP/missense	0.194 (0.065)	C/T	58/14
				CC/CT/TT	25/8/3

rs761386	Intron between Exons 9 and 10	SNP	0.222 (0.103)	C/T	56/16
				CC/CT/TT	22/12/2

rs5875654	Intron between Exons 10 and 11	InDel	0.222 (0.146)	8/7 GA	56/16
				88/78/77 GA	22/12/2

MAF = minor allele frequency. Bracketed values refer to MAF from HapMap-CHB populations or 1000 Genomes.

STR = short tandem repeat.

**Table 3:** Quantitative trait loci analysis of *GLP1R* gene variations with clinical response to the GLP-1 analogue by multiple linear regression analyses using additive genetic models with or without adjustment of co-valuables, including age, sex, BMI, DM duration, and glycemic states at baseline.

Gene variants (genotypes)	Clinical variables	Without adjustment		With adjustment	
		Coefficient (95% CI)	*P*	Coefficient (95% CI)	*P*
rs3765467 (CC, CT, TT)	Mean glucose_baseline	−1.604 (−18.944, 15.737)	0.852	−0.161 (−19.825, 19.502)	0.987
	Treatment	4.091 (−8.100, 16.282)	0.500	14.353 (4.202, 24.504)	0.007
	Change	−5.695 (−21.292, 9.902)	0.463	−14.514 (−32.324, 3.295)	0.106
	MAGE_baseline	−7.464 (−28.355, 13.427)	0.473	−8.870 (−31.366, 13.626)	0.427
	Treatment	5.453 (−15.296, 26.202)	0.597	13.222 (−10.084, 36.528)	0.255
	Change	−12.917 (−34.013, 8.179)	0.222	−22.092 (−46.635, 2.451)	0.076
	SDPG_baseline	−5.421 (−16.067, 5.224)	0.308	−7.647 (−19.858, 4.564)	0.210
	Treatment	6.073 (−3.538, 15.684)	0.208	10.588 (0.779, 20.398)	0.035
	Change	−11.494 (−21.161, −1.828)	0.021	−18.236 (−29.143, −7.328)	0.002

rs761386 (CC, CT, TT)	Mean glucose_baseline	−9.257 (−27.350, 8.835)	0.306	−2.968 (−22.396, 16.460)	0.757
	Treatment	−7.850 (−20.561, 4.861)	0.218	−1.868 (−13.249, 9.513)	0.739
	Change	−1.407 (−18.054, 15.240)	0.865	−1.100 (−19.545, 17.346)	0.904
	MAGE_baseline	0.828 (−21.470, 23.126)	0.940	7.109 (−15.240, 29.459)	0.520
	Treatment	−6.806 (−28.748, 15.137)	0.533	−7.774 (−31.184, 15.635)	0.502
	Change	7.634 (−15.061, 30.329)	0.499	14.884 (−10.156, 39.923)	0.234
	SDPG_baseline	8.493 (−2.571, 19.557)	0.128	10.451 (−1.319, 22.222)	0.080
	Treatment	−3.737 (−14.079, 6.605)	0.468	−3.083 (−13.510, 7.344)	0.550
	Change	12.230 (1.998, 22.462)	0.021	13.534 (1.826, 25.243)	0.025

SDPG: standard deviation of plasma glucose.

MAGE: mean amplitude of glycemic excursions.

CI: confidence interval.

**Table 4:** Quantitative trait loci analysis of *GLP1R* gene variations with 75 g OGTT response to the GLP-1 analogue by multiple linear regression analyses using additive genetic models with or without adjustment of co-valuables, including age, sex, BMI, DM duration, and glycemic states at baseline.

Gene variants (genotypes)	75 g OGTT variables	Without adjustment		With adjustment	
		Coefficient (95% CI)	*P*	Coefficient (95% CI)	*P*
rs3765467 (CC, CT, TT)	Glucose_0 minutes	−2.969 (−21.346, 15.409)	0.743	3.705 (−17.762, 25.172)	0.724
	30 minutes	−1.500 (−40.962, 37.962)	0.938	3.210 (−47.712, 54.132)	0.897
	60 minutes	2.250 (−53.419, 57.919)	0.935	5.139 (−66.086, 76.364)	0.883
	90 minutes	−15.344 (−69.476, 38.788)	0.566	−19.554 (−89.583, 50.475)	0.569
	120 minutes	−31.094 (−80.241, 18.053)	0.206	−38.048 (−100.420, 24.324)	0.220
	C-peptide_0 minutes	−0.151 (−1.102, 0.800)	0.747	−0.029 (−1.064, 1.007)	0.955
	30 minutes	−0.085 (−1.149, 0.978)	0.871	−0.021 (−1.185, 1.142)	0.970
	60 minutes	−0.521 (−1.821, 0.779)	0.419	−0.336 (−1.774, 1.101)	0.633
	90 minutes	−0.457 (−2.379, 1.466)	0.630	−0.584 (−2.807, 1.638)	0.592
	120 minutes	−1.257 (−3.677, 1.164)	0.297	−1.392 (−4.231, 1.447)	0.321
	Insulin_0 minutes	−17.459 (−81.946, 47.027)	0.584	−1.133 (−83.148, 80.882)	0.977
	30 minutes	−18.566 (−82.716, 45.585)	0.558	−2.694 (−84.957, 79.569)	0.947
	60 minutes	−22.191 (−87.217, 42.835)	0.490	−3.985 (−87.257, 79.287)	0.922
	90 minutes	−26.144 (−105.761, 53.474)	0.507	−6.281 (−108.580, 96.019)	0.900
	120 minutes	−34.109 (−121.846, 53.627)	0.433	−12.734 (−125.719, 100.251)	0.818

rs761386 (CC, CT, TT)	Glucose_0 minutes	−1.435 (−19.160, 16.290)	0.869	1.141 (−18.552, 20.834)	0.906
	30 minutes	12.957 (−24.721, 50.634)	0.704	10.326 (−36.077, 56.729)	0.650
	60 minutes	20.174 (−32.875, 73.222)	0.443	10.244 (−54.816, 75.304)	0.748
	90 minutes	20.826 (−30.997, 72.650)	0.417	18.288 (−45.775, 82.352)	0.561
	120 minutes	23.826 (−24.025, 71.677)	0.316	36.804 (−20.040, 93.649)	0.194
	C-peptide_0 minutes	−0.083 (−1.000, 0.834)	0.854	0.044 (−0.904, 0.991)	0.925
	30 minutes	0.129 (−0.894, 1.152)	0.798	−0.073 (−1.137, 0.991)	0.889
	60 minutes	0.519 (−0.732, 1.770)	0.403	0.159 (−1.162, 1.480)	0.806
	90 minutes	0.542 (−1.305, 2.390)	0.553	−0.022 (−2.069, 2.024)	0.982
	120 minutes	0.953 (−1.396, 3.302)	0.413	0.375 (−2.276, 3.026)	0.773
	Insulin_0 minutes	−27.735 (−89.251, 33.781)	0.364	−25.685 (−99.916, 48.546)	0.481
	30 minutes	−21.800 (−83.393, 39.793)	0.474	−22.497 (−97.156, 52.161)	0.539
	60 minutes	−17.687 (−80.485, 45.111)	0.569	−18.393 (−94.197, 57.411)	0.620
	90 minutes	−20.765 (−97.642, 56.111)	0.584	−21.338 (−114.531, 71.856)	0.640
	120 minutes	−21.678 (−106.715, 63.358)	0.606	−24.492 (−127.466, 78.483)	0.627

OGTT: oral glucose tolerance test.

DM: diabetes mellitus.

BMI: body mass index.

**Table 5:** Genetic variations of *GLP1R* studied in published experiments.

dbSNP rs# cluster ID	Region	Chromosome position^*^	Heterozygosity	MAF	Function
rs6923761	Exon 4	39065819	0.121	0.0647	Missense/homozygotes for the major allele associated with increase in GLP-1 response

rs3765467	Exon 5	39066296	0.260	0.1538	Missense/heterozygotes for the minor allele associated with increase in GLP-1 response

rs367543060	Exon 5	39066240	N.D.	N.D.	Missense/reduced GLP-1 response in *GLP1R* variant

^*^Chromosome position determined by GRCh38 assembly.

MAF: minor allele frequency.

N.D.: not determined.
