# Supplementary material for: Polymorphisms of GLP-1 Receptor Gene and Response to GLP-1 Analogue in Patients with Poorly Controlled Type 2 Diabetes
Source: J Diabetes Res. 2015 Feb 15;2015:176949. doi: 10.1155/2015/176949 (PMC4345081; doi:10.1155/2015/176949)
Supplement: Supplementary file 1 — The promoter, all 13 exons and intron-exon boundaries of the GLP1R gene (GenBank accession number AL035690) were amplified by polymerase chain reaction (PCR) using specific primer sets in Supplementary Table 1. The Titanium Taq DNA Polymerase (Clontech Laboratories, Inc., Mountain View, CA, USA) was used in PCR. The PCR amplification conditions consisted of initial denaturation at 95°C for 5 minutes followed by 40 cycles at 95°C for 45 seconds, annealing temperature for 45 seconds and 72°C for 45 seconds, with one additional cycle at 72°C for 10 minutes. [file 176949.f1.zip › Supplement _Table 1_Primer design.doc]

|  | Forward primer  (5′ → 3′) | Reverse primer  (5′ → 3′) | Position* | Segment size (bp) | Annealing  temperature (°C) |
| --- | --- | --- | --- | --- | --- |
| Promoter | TCAGGCCTCAGTTTCCTTTG | CAGGGTCCCGGTTCTTCC | chr6:39016191+39016488 | 298 | 50 |
| Exon |  |  |  |  |  |
| 1 | TCCTGCGCTTCCTGATTC | GTCCCTGGATCTCACCTG | chr6:39016358+39016709 | 352 | 50 |
| 2 | ACAGGCCTCCCATATATG | CATCAAAGTCCTCCAGAAGTT | chr6:39024143+39024335 | 193 | 50 |
| 3 | ACAAGCAGGGACTCAGAGACTG | TACCAGGGGCAGCTGACATTC | chr6:39025210+39025335 | 126 | 55 |
| 4 | GCGTATATGTCAGGGGAGGA | TTTGTCCAGAAAGCATGGTG | chr6:39033286+39033760 | 475 | 57 |
| 5 | CATGGGAAGGAAGATTGTGG | AAAGCTCCAAAGGGAGGAAG | chr6:39033902+39034485 | 584 | 57 |
| 6 | AAGAAGACACACGCACAGTC | TCGGCTCTTGTCTTCTCCTG | chr6:39040512+39040893 | 382 | 57 |
| 7 | CCTAGCCAGAGATGTGAGCT | CAGCAGTGCGTTCCAAGTAC | chr6:39041252+39041636 | 385 | 57 |
| 8 | TCGGCATGTAGACTGTGGCTC | AAGGAATCCCATGGACCTTG | chr6:39046032+39046225 | 194 | 55 |
| 9, 10 ^†^ | GGACTATGCGTGTGTGTTGTG | TTTCTCTACCCTCCCCACTTC | chr6:39046535+39047279 | 745 | 57 |
| 11^†^ | AACCCTTGCTTTTGCCCTGTCT | GCTTCTTGTCCCATGTGCTTAC | chr6:39047121+39047694 | 574 | 57 |
| 12 | AACCCTGGTGTTCCTTCAGA | TGTGGCTTGGGTATGTGTGT | chr6:39048289+39048861 | 573 | 55 |
| 13 | ATGATGGCTTTCGTTTCCCTCC | CTGGAGTCTCAGCTGCAGGAGG | chr6:39053646+39053857 | 212 | 50 |

Supplement - Table 1. Nucleotide sequences of DNA primers used for PCR amplification of the GLP-1 receptor (*GLP1R*) gene

* Each position is expressed according to the sequence registered in Human Feb. 2009 (GRCh37/hg19) Assembly.

^†^Reference 20
